# Characterizing Engagement with Web-Based Screening, Brief Intervention, and Referral to Treatment (SBIRT) for Traumatic Stress and Substance Misuse After Interpersonal Violence

**DOI:** 10.3390/ijerph22020190

**Published:** 2025-01-29

**Authors:** Alexandra N. Brockdorf, Emily L. Tilstra-Ferrell, Carla K. Danielson, Angela D. Moreland, Alyssa A. Rheingold, Selime R. Salim, Amanda K. Gilmore, Rachel E. Siciliano, Daniel W. Smith, Christine K. Hahn

**Affiliations:** 1 Department of Psychiatry and Behavioral Sciences, Medical University of South Carolina, 67 President Street, Charleston, SC 29425, USA; ferrelle@musc.edu (E.L.T.-F.); danielso@musc.edu (C.K.D.); moreland@musc.edu (A.D.M.); rheingaa@musc.edu (A.A.R.); salims@stanford.edu (S.R.S.); siciliar@musc.edu (R.E.S.); smithdw@musc.edu (D.W.S.); hahnc@musc.edu (C.K.H.); 2 Department of Psychiatry and Behavioral Sciences, Stanford University, 291 Campus Drive, Li Ka Shing Building, Stanford, CA 94305, USA; 3 School of Public Health, Georgia State University, Atlanta, GA 30302, USA; agilmore12@gsu.edu

**Keywords:** violence, substance-related disorders, posttraumatic stress disorder, trauma, brief interventions, alcohol treatment, health screening

## Abstract

Screening, brief intervention, and referral to treatment (SBIRT) is a widely used public health approach for delivering early intervention for substance misuse. SBIRT adaptations that incorporate content on interpersonal violence and posttraumatic stress disorder (PTSD) symptoms may be warranted, as experiences of interpersonal violence are prevalent and associated with greater substance misuse; however, more research is needed to refine the delivery of PTSD-substance use content within the SBIRT model. This study examined clinical data collected as part of a web-based SBIRT developed for co-occurring substance misuse and PTSD symptoms after interpersonal violence to characterize the clinical symptoms and responses of adults presenting to agencies serving intimate partner and sexual violence survivors. The respondents (*N* = 52) completed self-report measures during the SBIRT tool to personalize the recommendations, as well as motivational enhancement exercises. Descriptive statistics were conducted. The results underscored high rates of probable PTSD, substance use, and trauma-related motives for substance use. The respondents were ready to change their substance use on average after receiving personalized feedback. Many expressed values related to trauma recovery and self-empowerment, perceived these values as useful for substance use reduction, and set goals to seek mental health services or reduce their drinking quantity. The findings point to several clinical targets for integrated PTSD-substance misuse interventions for interpersonal violence survivors.

## 1. Introduction

Interpersonal violence, referring to the intentional use of force against another person or community with a high likelihood of harm, is prevalent and concerning [1]. Two significant forms of interpersonal violence are intimate partner violence and sexual violence. National estimates indicate that nearly half of women and men in the United States have experienced lifetime sexual violence, physical violence, and/or stalking by an intimate partner [2]; further, transgender and gender-nonbinary people are 1.7 times more likely to experience intimate partner violence during their lifetime compared to cisgender people [3]. In addition, over half of women and almost a third of men have experienced unwanted sexual contact in their lifetime [4]. Unfortunately, these forms of trauma exposure are associated with wide-ranging psychological, physical health, and economic consequences for the survivors, including a greater risk for posttraumatic stress disorder (PTSD) and substance misuse [5,6,7,8]. Heightened substance use following trauma exposure has been largely attributed to the self-medication model, which posits that survivors use substances to cope with PTSD symptoms [9,10,11]. Indeed, intimate partner violence survivors with PTSD are nearly seven times more likely to use drugs and fifteen times more likely to use both alcohol and drugs on a given day compared to those without PTSD [12]. Among women receiving substance use treatment, interpersonal violence exposure is indirectly associated with opioid and cocaine use and alcohol misuse via PTSD symptoms [13]. Because substance misuse is associated with adverse mental and physical health outcomes [14,15,16], as well as high economic costs [17], reducing substance misuse after interpersonal violence exposure is an important public health aim.

One increasingly common avenue for the prevention and treatment of substance misuse is brief intervention, which is intended to be a scalable, accessible method for reaching larger proportions of the population. Many brief interventions for substance misuse are designed to be delivered by a wide range of health professionals across diverse settings (e.g., primary care, emergency departments, schools) or via online methods for increased accessibility [18,19,20]. Brief interventions can also be broadly disseminated to provide greater opportunities for the early detection and treatment of substance misuse. Because of these strengths, screening, brief intervention, and referral to treatment (SBIRT) is a widely used public health approach to identify and deliver early intervention for substance misuse [21]. SBIRT begins with the administration of brief, validated screening measures to assess current alcohol and drug use and misuse [22,23]. Individuals whose scores place them at risk for misuse then receive a brief intervention. Brief interventions vary in content [24], but often include components such as psychoeducation (e.g., recommended limits for alcohol consumption, blood alcohol content [BAC], alcohol and drug use consequences), personalized normative feedback, motivational interviewing strategies focused on facilitating readiness to change, and goal-setting activities (e.g., selecting harm-reduction strategies, identifying social support). Finally, individuals who are using drugs and/or drinking at more severe levels are offered resources and referrals for alcohol and substance use disorder treatment, which may include outpatient and/or inpatient services to help individuals safely reduce their use [22]. Importantly, the personalized nature of SBIRT allows for tailored intervention content and referrals based on each person’s responses.

There is strong empirical support for the efficacy of the screening and brief intervention components of SBIRT, with less support for referral to treatment [20,21,25]. Meta-analytic evidence indicates that brief alcohol interventions led to greater reductions in the average weekly alcohol use quantity at follow-up periods ranging from six to twelve months compared to control conditions [23]. Across 17,575 patients who received SBIRT services, alcohol use, heavy drinking, and substance use were significantly reduced six months post-SBIRT compared to the pre-SBIRT levels [26]. Accordingly, the US Preventive Services Task Force recommends screening and brief intervention for alcohol misuse among adults in primary care [27]. There is also increasing evidence supporting SBIRT as an effective strategy for reducing drug use [22], although more research examining its efficacy for specific substances is needed [28].

Given the strong evidence base supporting the use of screening and brief intervention to identify and reduce substance misuse, there may be benefits to tailoring SBIRT specifically for interpersonal violence survivors. Some research indicates that interpersonal violence is most robustly associated with the development of substance misuse and PTSD compared to other trauma types [29,30,31], suggesting the need for targeted substance misuse interventions for individuals who have experienced intimate partner and sexual violence. Tailored programs could also help address barriers that might limit SBIRT uptake and engagement among interpersonal violence survivors [32]. For instance, many intimate partner and sexual violence survivors report feeling ashamed of their use of alcohol to cope with distress after violence and worry about being blamed for their drinking [32]. Nonjudgmental efforts to address substance use in the context of interpersonal violence, such as noting how substance use may have changed after violence and may be driven by efforts to alleviate distress, may be particularly empowering for this population. Because web-based programs typically have less or no provider integration, this type of program delivery could further reduce fears about being blamed or dismissed by healthcare providers [33].

Another important part of tailoring SBIRT for interpersonal violence survivors may be addressing related concerns that are frequently comorbid with substance misuse, such as PTSD [29,34,35]. Because of this high comorbidity, integrated psychological treatments that concurrently address substance misuse and PTSD symptoms are increasingly favored [36]. Further, many individuals do not seek treatment for their PTSD symptoms [37], suggesting that adapting SBIRT to include screening for trauma exposure and PTSD symptoms could help facilitate broader access to trauma-focused psychoeducation and intervention through universal screening. The initial evidence supports the application of SBIRT for trauma exposure and PTSD symptoms [38,39]. For instance, patients were highly satisfied with a trauma-focused SBIRT and subsequently increased their use of behavioral health services [38]. Similarly, a sequential trauma-focused SBIRT that was administered directly after a traditional substance-focused SBIRT demonstrated a high level of acceptability [39]; further, almost two-thirds of patients accepted a mental health referral at the end of the trauma-focused SBIRT, with the highest rates of acceptance occurring among those who screened positive for both PTSD and alcohol misuse [39]. These high rates of accepted referrals suggest that tailoring SBIRT could help enhance the efficacy of referral to treatment [25]. However, these programs have not yet addressed the specific type of trauma exposure, despite the likelihood of unique barriers and considerations among individuals who have experienced interpersonal violence [32,33].

To address these barriers, Hahn et al. [40] developed a web-based SBIRT for traumatic stress and substance use following interpersonal violence (Choices for your Health After Trauma, or “CHAT”). CHAT was designed to have a high degree of personalization to facilitate survivors’ agency and empowerment in making healthy choices about their substance use, which many survivors noted as a strength [40]. Given these opportunities for personalization, the responses on CHAT could help reveal brief intervention strategies that are particularly appealing to interpersonal violence survivors and, therefore, inform key components of future programming. The present study describes the responses during CHAT across three primary domains—clinical symptoms, readiness to change, and selections during motivational exercises aimed at facilitating reduced substance use—with the goal of informing the development and refinement of integrated PTSD-substance use SBIRT interventions for interpersonal violence survivors.

## 2. Materials and Methods

### 2.1. Participants and Procedures

The SBIRT tool (Choices for your Health After Trauma, or “CHAT”) was created on REDCap, a secure, web-based application that supports electronic data capture [41]. CHAT is based on previous SBIRT programs for trauma survivors [40]. CHAT involves several main components. First, responders select whether they would prefer to view content for women, men, or general/gender-neutral content. Second, CHAT shares psychoeducation on alcohol and drug use in relation to interpersonal violence. The third component of CHAT involves a self-report screening of alcohol and drug use (e.g., severity, motives, consequences) and PTSD symptoms, as well as the selection of important personal values. Fourth, piping logic based on each person’s responses is used to provide personalized feedback on drinking quantity and frequency, the National Institute on Alcohol Abuse and Alcoholism (NIAAA)-recommended drinking limits, alcohol and drug use consequences, and PTSD symptoms. Respondents who reported low-risk substance use were given prompts that encouraged low use (e.g., “Based on your responses, your drinking is below the “at risk” level. Keep up the good work!”). Fifth, the respondents completed several motivational exercises aimed at facilitating and supporting healthy substance use, such as connecting their readiness to change with the personal values they selected earlier in CHAT, setting goals for their substance use, and identifying social support and coping skills that they can use to aid substance use reduction (or the maintenance of their low-risk use as appropriate). Finally, they received a personalized plan summarizing their intervention choices and content, as well as local referrals for mental health treatment, substance use treatment, and violence advocacy organizations.

CHAT was iteratively developed and refined using feedback from survivors of intimate partner and sexual violence and victim service professionals at local advocacy centers in the US Southeast. The results from interviews and focus group discussions during and after viewing CHAT revealed that survivors and providers perceived CHAT as easy to use, appropriate for themselves or their patients, and beneficial for addressing trauma and substance use [40]. CHAT was then disseminated to several local agencies serving interpersonal violence survivors: a non-profit advocacy center serving primarily intimate partner violence survivors, a non-profit advocacy center serving primarily sexual assault survivors, the region’s sexual assault nurse examination (SANE) program, and a mental health research and treatment program that provides trauma-focused psychotherapy. The research team conducted brief training on the use and delivery of CHAT with staff and service professionals at each agency. The providers were informed that CHAT is a self-directed, web-based intervention for substance use after interpersonal violence. The research team reviewed the core components of CHAT, discussed possible scenarios for using CHAT, answered any questions, and provided the REDCap link. The providers were instructed to share the link for CHAT with interpersonal violence survivors presenting at each agency who might benefit from substance use education and intervention. All the procedures were approved by the IRB; informed consent was waived due to minimal risk.

Fifty-two respondents engaged with CHAT. Because CHAT was distributed anonymously and directly to survivors by service professionals based on feedback indicating that privacy was a priority for the survivors and providers [40], respondent demographic and trauma exposure data were not collected. However, 75.0% of the respondents indicated that they wanted to view content for women, 3.8% selected content for men, 15.4% selected general content, and 5.8% indicated that they preferred not to respond (which was piped to provide general content). Most respondents (*n* = 42, 80.7%) reported consuming alcohol and 51.9% (*n* = 27) reported the lifetime use of drugs other than those required for medical reasons. Among the respondents who reported lifetime drug use, 44.4% (*n* = 12) reported use of one drug class, 51.9% reported polysubstance use (*n* = 14), and 3.7% (*n* = 1) did not indicate the specific substances they had used. The specific drugs used can be found in Table 1.

### 2.2. Measures

An overview of the measures used in this study are provided below in Table 2.

#### 2.2.1. Symptom Measures

Self-report measures on substance use and PTSD symptoms were included within CHAT to provide the basis for personalized feedback. The following measures were used to characterize the sample.

PTSD symptoms were assessed using the Primary Care PTSD Screen for DSM-5 (PC-PTSD-5), which is a 5-item self-report screening measure for PTSD [42]. Two items assess alterations in mood and cognition, and the remaining three items assess intrusions, avoidance, and hyperarousal. The respondents rated whether they experienced each symptom on a dichotomous scale (*yes* = 1, *no* = 0). The items were summed. Consistent with prior research identifying 4 as the most efficient cut-off score [43], the total score was recoded so that scores above the cut-off score (i.e., 4 or 5) were recoded to 1, reflecting a positive PTSD screen, and the remaining scores (i.e., 0 to 3) were recoded to 0, reflecting a negative PTSD screen. The PC-PTSD-5 demonstrates good agreement with clinician-administered structured interviews for PTSD [43].

Substance use motives were assessed using a 14-item measure (“What are some of your reasons for using substances?”) The items were created for CHAT by adapting similar measures from other SBIRT models and motivational models of substance use [44,45]. The respondents selected *yes* (1) or *no* (0) according to their belief about whether they used the substances for the reason specified in each item.

A single item from the Drug Abuse Screening Test (DAST) [46] was used to assess lifetime drug use: “In your lifetime, have you used drugs other than those required for medical reasons?” The respondents indicated either *yes* (1) or *no* (0).

Alcohol consumption levels were assessed using the Alcohol Use Disorders Identification Test—Consumption (AUDIT-C), a 3-item measure of alcohol consumption that is well supported as a screening measure for probable alcohol misuse [47]. The items were summed, with a possible range of 0 to 12. Because the cut-off scores for the AUDIT-C are based on sex (i.e., a score of 3 indicates hazardous drinking for individuals assigned female at birth and a score of 4 indicates hazardous drinking for individuals assigned male at birth) and we did not assess sex or gender identity, we did not use the clinical cut-off scores. Higher scores indicated a greater risk of alcohol misuse.

Substance use changes after violence were assessed using two items. Specifically, “How has your alcohol use changed since your most recent experience with violence?” was assessed if the respondents endorsed alcohol use and “How has your drug use changed since your most recent experience with violence?” was assessed if the respondents endorsed lifetime drug use. The response options were *I have increased my alcohol/drug use* (1), *I have decreased my alcohol/drug use* (2), and *there has been no change in my alcohol/drug use* (3). The responses were then dichotomized to indicate whether the substance use increased or did not increase.

#### 2.2.2. Readiness to Change and Motivational Exercises

After reviewing their psychoeducation and personalized feedback, the respondents indicated their readiness to change. The single-item readiness ruler was used to assess readiness to change [48] on a visual analog scale ranging from 0 (*not at all ready to change*) to 100 (*fully ready to change*). The readiness ruler predicts decreased use of drugs [49], as well as fewer drinks consumed per drinking days and days without drinking [50], supporting the item’s predictive validity. The respondents indicated their readiness to change any aspect of their substance use after receiving psychoeducation and personalized feedback on their substance use and PTSD symptoms. The respondents only completed the readiness ruler if they reported potentially hazardous levels of alcohol or drug use based on the endorsement of at least one of the following criteria: reporting having at least three to four drinks on a typical drinking day on the AUDIT-C, reporting consuming six or more drinks in a single episode on the AUDIT-C, endorsing any drug use on the DAST, or reporting having increased substance use since their most recent experience of violence. Four respondents did not meet any of these criteria, and thus, did not complete the readiness ruler.

At the start of the screening portion of CHAT, the respondents were asked to indicate the principles, standards, or qualities that they considered most important in their life. The respondents could select from a list of 15 values (e.g., achievement: to have accomplishments I can be proud of), as well as a 16th “other” category, with the option to describe a value that was not listed. After the respondents indicated their readiness to change during the brief intervention portion of CHAT, they were asked to indicate how each value they selected related to choosing their expressed ruler number (i.e., readiness to change) instead of choosing a lower number. They were then asked one of two items focused on either changing misuse or maintaining healthy use for each selected value. If their AUDIT-C scores placed them at risk for alcohol misuse or if they reported using drugs, they received a prompt focused on changing their substance use (e.g., “Changing my substance use would help me achieve things in my life that I could feel proud of”). If their AUDIT-C scores did not place them at risk for alcohol misuse and they reported not using drugs, they received a prompt focused on maintaining healthy use (e.g., “Making healthy choices about substances use helps me to achieve things in my life that I could feel proud of”). The response options included *I agree*, *I disagree*, and *I am not sure*.

The respondents were then asked to identify one goal related to their substance use from a list of options: “We encourage you to pick ONE goal that you feel confident you can accomplish”. The respondents could select from several options, including substance use goals, substance use and mental health service utilization, describing another goal, or declining to set a goal. The specific substance use goals varied based on the responses to the screening measures. The respondents who reported alcohol and drug use could select goals regarding a reduction in the use of either substance, those who reported only alcohol use could select goals regarding a reduction in alcohol use, those who reported only drug use could select goals regarding a reduction in drug use or continued alcohol abstinence, and those who reported no substance use could select goals regarding continued abstinence.

The respondents reviewed the rationale for behavioral activation and selected from 15 possible options: “Doing healthy things is an important part of recovering from violence. What are THREE activities you can try? Pick things you like to do and are easy for you”. The last option allowed the respondents to identify other activities that were not listed.

## 3. Results

Descriptive statistics, including means (*SD*) and frequencies (*n*), were calculated in SPSS to characterize the sample and their responses to the brief intervention exercises.

### 3.1. Sample Characteristics

Of the 52 respondents, 29 (55.8%) completed CHAT and the remaining 23 respondents discontinued the tool prior to completion. Most respondents (80.8%, *n* = 42) screened positive for PTSD on the PC-PTSD-5. The average respondent was drinking at levels suggestive of alcohol misuse (*M* = 5.0, *SD* = 3.5) by both of the clinical cut-offs based on male and female sex. Approximately half of the respondents (51.9%, *n* = 27) reported lifetime use of drugs other than those required for medical reasons. Over half of the 42 respondents who reported alcohol use indicated that their alcohol use increased after their most recent experience of violence (59.5%, *n* = 25) and less than half of the 27 respondents who reported drug use indicated that their drug use increased after violence (40.7%, *n* = 11). The most common motives for substance use were all related to coping with trauma symptoms: to relax; to deal with feeling overwhelmed; to try and get rid of feelings like guilt, sadness, or anger; to cope; and to forget about things that have happened to them (Table 3).

### 3.2. Motivational Exercises

The respondents were ready to change their substance use on average, with a mean of 76.9 (*SD* = 24.1, range: 0–100). Regarding personal value selection during CHAT (Table 4), the respondents selected moving forward/healing from past events, mental health, and taking care of their children most frequently. The respondents unanimously agreed that their values of religion, healing, self-reliance, and spirituality were helpful for substance use maintenance and reduction. In contrast, the values of friendship, self-image, and love had the lowest rates of agreement regarding perceived usefulness for substance use change. Regarding personal goal selection (Table 5), the respondents most often selected the goal of talking to a provider about their mental health, followed by drinking fewer drinks than their typical amount, stopping the consumption of six or more drinks during a single drinking episode, and stopping alcohol use completely. No one selected the goals of drinking less frequently than their typical drinking pattern or continuing to not use drugs. Only two respondents did not want to set a goal. Finally, the respondents selected the following pleasurable activities most often: taking a walk, contacting a support person they identified in CHAT or another friend/family member, and watching television or a movie (Table 6). The respondents were least likely to write in their own activity or select that they would play video games, look at pictures of their loved ones, or go to the mall.

## 4. Discussion

The development of brief interventions for substance misuse and PTSD among interpersonal violence survivors is sorely needed. The findings from this study indicate that the respondents reported high levels of PTSD symptoms, the use of substances to cope with distress, and alcohol and drug use; they were also ready to change on average, underscoring the potential relevance of brief intervention. The respondents selected an array of individual values, goals, and pleasurable activities, indicating the need to personalize intervention content and clinical recommendations to incorporate individual survivor preferences. The findings suggest that trauma-related motives for substance use, as well as values and goals centered around trauma recovery, may be particularly relevant clinical targets for survivors of interpersonal violence such as intimate partner violence and sexual assault.

Supporting the well-documented comorbidity between PTSD and substance misuse [29,34,35], the rates of probable PTSD, alcohol misuse, and lifetime drug use were all high among our sample and the respondents frequently reported that they were motivated to use substances to help them escape or alleviate trauma symptoms. The findings indicate that individuals presenting to community agencies serving intimate partner and sexual violence survivors are likely to be using substances at potentially hazardous levels and perceive trauma symptoms as an important contributor to alcohol and drug use. Addressing the connection between PTSD symptoms and substance misuse may be a critical part of psychoeducation and intervention for interpersonal violence survivors. CHAT addresses this connection in several ways, including sharing psychoeducation on the prevalence of substance use before interpersonal violence, as well as trauma-related drinking motives and how the use of substances to cope with difficult emotions can lead to negative consequences over time. In addition, values related to trauma recovery (e.g., mental health and healing after trauma, reclaiming self-esteem and independence) were predominantly perceived as helpful for substance use reduction, suggesting that it may be beneficial to highlight these values when working with survivors. For instance, brief interventions could frame substance use changes as a strategy for empowerment after interpersonal violence, as well as note the benefits of reducing alcohol and drug use for trauma recovery and mental health.

However, it is notable that the respondents identified an array of reasons underlying substance use in addition to coping-oriented motives. Social motives were frequently endorsed, including using substances to celebrate with others or facilitate social interactions, or because others around them use substances. The respondents were mixed as to whether values related to social relationships would benefit their efforts to achieve or maintain low levels of substance use. This variability could reflect differences in substance use within social networks, such as if people are less likely to perceive social values as helpful when substance use is a common shared activity with friends or family. Similarly, survivors may be less ready to reduce their use if they are in intimate relationships where their partner(s) are drinking or using drugs at similarly high levels, as substance use concordance is associated with a better relationship quality [51]. These social motives and settings may be particularly relevant for revictimization risk among interpersonal violence survivors, such as if survivors are using substances with intimate partners who have previously been violent or are drinking in settings with exposure to potential perpetrators (e.g., bars, parties). Interventions to reduce substance misuse may benefit from assessing the presence of close others who are drinking and/or using drugs and providing normative feedback based on larger population norms. Because reaching out to social support was the second most selected pleasurable activity, interventions could also help survivors identify close others who can support their goals for alcohol and/or drug use reduction, which could include peer support groups [52]. For some survivors, this could also include religious support to aid recovery [53]; although religion was the least commonly selected personal value, it was perceived as helpful when it was selected.

Our findings indicate that the respondents made highly individualized choices regarding their preferred strategies to support substance use reduction, supporting the use of motivational interviewing techniques in CHAT. The wide range of selected personal values and strategies during CHAT supports the integration of individual preferences into brief interventions and is consistent with the larger focus on patient characteristics in the evidence-based practice model. All the respondents who answered the goal-selection question except two were ready to set a goal, which may reflect the intervention effectiveness or that the respondents who completed SBIRT were already motivated to make changes to their substance use. The goal that the respondents selected most frequently was talking to a service provider about their mental health, which was selected over three times as often as talking to a provider about their substance use; this suggests that there may be potential benefits in integrating providers into the web-based intervention. Respondents may be highly motivated to address trauma-related distress, which could represent an entry point to mental health service utilization. The prevalence of this selected goal could also reflect desire for integrated treatment that considers multiple aspects of psychological functioning, rather than a sole focus on substance use [36]. Further, concurrent PTSD treatment may bolster the treatment for substance misuse, as greater PTSD symptom severity predicts more frequent substance use following residential substance use disorder treatment [54]. Another interesting finding is that the respondents were more likely to select goals related to reducing their drinking quantity versus drinking frequency. It is possible that survivors view drinking quantity as particularly associated with negative consequences (e.g., hangover symptoms, next-day impairment) and, therefore, a more impactful target for intervention.

Finally, the respondents selected an array of pleasurable activities, ranging from physical activity (e.g., walking, exercising) to social support (e.g., calling a friend, spending time with pets), distraction (e.g., watching television, taking a drive), and relaxation (e.g., meditation). These findings indicate that patients can likely identify multiple types of enjoyed activities that could replace substance use and facilitate behavioral activation, despite experiencing PTSD symptoms such as a loss of interest and a depressed mood.

### Limitations and Future Directions

The present study had several strengths, including the examination of clinical data from a novel web-based SBIRT program disseminated to agencies that serve interpersonal violence survivors and a sample of respondents with high rates of PTSD symptoms and substance misuse, but there are also important limitations to consider. Because CHAT did not include demographic measures, we do not know the demographic characteristics or interpersonal violence history of the respondents who used CHAT, which limits our understanding of the study’s generalizability and how respondent characteristics might have influenced the values, goals, and activities that were selected during CHAT. We also had a small sample size of 52 individuals who predominantly selected content for women. Replication is an important next step to see if similar descriptive patterns emerge in larger samples with gender and cultural diversity.

Although many respondents selected personal goals and pleasurable activities, it is possible that the respondents did not implement these plans after completing CHAT. Future studies should implement follow-up assessments after SBIRT completion to evaluate the adherence and effectiveness over time. Such an examination is especially crucial in light of prior findings that SBIRT did not increase alcohol treatment utilization across nine randomized controlled trials [25], suggesting the need for the continued evaluation and implementation of referral to treatment efforts. There may be remaining barriers to treatment that could be targeted through continued follow-up with survivors after the initial SBIRT. Moreover, almost half of the respondents discontinued the SBIRT and did not complete all modules. This was a surprising finding, given that CHAT had a high level of acceptability with survivors during its formative evaluation [40]. A further assessment of the barriers to SBIRT completion, including through the use of qualitative methods, would help clarify the reasons for early drop-out. For instance, it is possible that CHAT was too lengthy or that the respondents would have been more likely to complete the SBIRT with increased provider engagement, such as completing CHAT or reviewing the personal plan together. Alternatively, CHAT may not have been relevant for all the respondents, such as if providers shared the link with survivors who were not interested in learning more about substance use after trauma. These limitations and questions are critical to address in future research to improve the development of CHAT and other brief, web-based interventions for interpersonal violence survivors.

Our results point to several future directions for the development and refinement of SBIRT for substance misuse after trauma. First, an evaluation of strategies to assess and enhance engagement in web-based intervention remains an important goal. Future work could compare readiness to change, motivational exercise selections, and engagement after completing the present version of CHAT versus a provider-enhanced version, such as through coaching phone calls, text messages, or assisted completion of the intervention. Future studies could also compare dismantled versions of CHAT to examine whether components could be eliminated to increase the brevity without compromising the intervention’s effectiveness. In addition, a continued evaluation of the dissemination and implementation of CHAT in community settings is an important future direction to maximize the treatment uptake and utilization, especially for survivors with greater barriers to the receipt of evidence-based treatment.

The findings from the present study also have implications for clinicians providing services to interpersonal violence survivors. The responses during CHAT indicate perceptions of PTSD symptoms and substance use as interconnected concerns, including the frequent use of substances as a strategy for coping with trauma-related distress. These perceptions support the relevance of integrated PTSD-substance misuse content within brief interventions and existing gold-standard treatments, such as Concurrent Treatment of PTSD and Substance Use Disorders Using Prolonged Exposure, or COPE [55]. Similarly, referral to treatment and motivational enhancement efforts may benefit from highlighting change talk around trauma-related mental health difficulties in light of the findings that the respondents selected goals focused on addressing their mental health more often than goals specific to their substance use. In addition, clinicians should seek to integrate patient values and preferences into treatment, including assessing how someone’s substance use is and is not consistent with their values and emphasizing treatment as a means of promoting healing and empowerment after interpersonal violence. Lastly, CHAT could be directly integrated within clinical care, such as being administered in sessions or used as a guide for providers when discussing substance use with patients.

## 5. Conclusions

In summary, these findings underscore the relevance of integrated interventions for substance misuse and PTSD symptoms following interpersonal violence. Most respondents indicated that their alcohol and/or drug use increased after violence exposure and endorsed trauma-related coping motives for substance use. Acknowledging the role of violence exposure and tailoring content to address each person’s reasons for use, as well as personalized protective values and activities, may be an important part of ensuring that web-based interventions are trauma-informed and relevant for interpersonal violence survivors. Although follow-up assessments are an important next step for future research, the respondents were ready to change on average after receiving brief intervention content and selected a variety of future goals and alternative activities to substance use. The findings from the present study suggest that values and goals related to mental health recovery after trauma may be helpful to incorporate into substance use treatment with survivors of intimate partner and sexual violence.

## Figures and Tables

**Table 1:** Rates of drug use.

Drug Use	*n* (%) of Past Year Use	*n* (%) of Past Three-Month Use
Any Endorsement	26 (50.0%)	23 (44.2%)
**Drug Type** ^1^		
Cannabis	26 (100%)	22 (95.7%)
Prescription stimulants	11 (42.3%)	9 (39.1%)
Sedatives or sleeping pills	10 (38.5%)	7 (30.4%)
Cocaine	8 (30.8%)	4 (17.4%)
Hallucinogens	8 (30.8%)	4 (17.4%)
Opioids	6 (23.1%)	3 (13.0%)
Methamphetamine	6 (23.1%)	3 (13.0%)
Inhalants	3 (11.5%)	1 (4.3%)

^1^ Drug type percentages represent the number of respondents who reported using each drug type out of those who reported any drug use in the past year or past three months.

**Table 2:** Overview of measures.

Measure	Number of Items	SBIRT Phase
Values	16	Screening
PTSD symptoms (PC-PTSD-5)	5	Screening
Substance use motives	14	Screening
Drug use (DAST)	1	Screening
Alcohol consumption (AUDIT-C)	3	Screening
Substance use change after violence	2	Screening
Readiness to change	1	Brief Intervention
Values in relation to readiness to change	Number of values selected during screening	Brief Intervention
Goals	1	Brief Intervention
Healthy activity selection	15	Brief Intervention

**Table 3:** Substance use motives.

Substance Use Motives	*n* (%) Selected
To relax	31 (59.6%)
To deal with feeling overwhelmed	30 (57.7%)
To try and get rid of feelings like guilt, sadness, or anger	28 (53.8%)
To cope	25 (48.1%)
To forget about things that have happened to me	21 (40.4%)
To celebrate with friends and/or family	17 (32.7%)
To make it easier to socialize	16 (30.8%)
It feels good	15 (28.8%)
To have fun	13 (25.0%)
My friends and/or family drink or use substances	12 (23.1%)
For taste	7 (13.5%)
I’ve had withdrawal symptoms and keep using so I don’t feel sick	2 (3.8%)
Other reasons (respondents described in text: to fit in with peers, to sleep, because of coercion or force, to reduce fear, to cope with grief)	2 (3.8%)
I don’t have any reasons to drink or use drugs	0 (0%)

**Table 4:** Perceived helpfulness of values in relation to substance use.

Value	*n* (%) Selected as a Personal Value	*n* (%) Perceived Value as Helpful for Substance Use
Moving forward: to heal from past events	31 (59.6%)	3 (100%) ^1^
Health (mind): to have good mental health	28 (53.8%)	15 (75.0%)
Children: to take good care of my children	20 (38.5%)	12 (85.7%)
Self-reliance: to be strong, capable, and independent	19 (36.5%)	1 (100%)
Self-image: to feel good about myself	18 (34.6%)	9 (64.3%)
Family: to have a happy, loving family	16 (30.8%)	10 (83.3%)
Achievement: to have accomplishments I can be proud of	13 (25.0%)	7 (77.8%)
Friendship: to have close, supportive friends	13 (25.0%)	5 (62.5%)
Health (body): to have good physical health	13 (25.0%)	7 (70.0%)
Love: to feel loved by others	11 (21.2%)	6 (66.7%)
Safety: to be safe and secure	9 (17.3%)	6 (85.7%)
Reputation: to be respected by others	8 (15.4%)	5 (71.4%)
Spirituality: to connect to something bigger than myself	8 (15.4%)	6 (100%)
Wealth: to have plenty of money	4 (7.7%)	3 (75.0%)
Religion: to practice my religion	1 (1.9%)	1 (100%)

^1^ Due to a piping error in the SBIRT tool, only the respondents who reported healthy levels of substance use responded to items assessing perceptions of healing and self-reliance related to substance use. The percentages in the third column correspond to the percentages of respondents who perceived the value as being helpful among those who responded to the item.

**Table 5:** Goal selection.

Goals	*n* (%) Selected ^1^
I will talk to a provider about my mental health.	10 (29.4%)
I will drink less than [typical number of standard drinks reported on the AUDIT-C] drinks when I drink.	5 (14.7%)
I will stop drinking more than six drinks at a time.	4 (11.7%)
I will stop using alcohol completely.	4 (11.7%)
I want to set my own goal (respondents described continuing the changes they have already made, seeking formal support).	3 (8.8%)
I will talk to my provider about my alcohol or drug use.	2 (5.9%)
I don’t want to set a goal right now.	2 (5.9%)
I will talk to my provider about my alcohol use.	1 (2.9%)
I will continue to not drink alcohol.	1 (2.9%)
I will stop using drugs completely.	1 (2.9%)
I will reduce the amount of drugs I use.	1 (2.9%)
I will continue not to use drugs.	0 (0.0%)
I will drink less than [typical drinking frequency reported on the AUDIT-C] when I drink.	0 (0.0%)

^1^ A total of 34 respondents completed the goal-selection activity. The options for specific substance use goals varied based on the responses to the screening measures.

**Table 6:** Pleasurable activity selection.

Activity	*n* (%) Selected ^1^
Take a walk	21 (55.3%)
Call or text [identified support person] or other friend or family member	18 (47.4%)
Watch TV or a movie	18 (47.4%)
Take a shower	15 (39.5%)
Play a sport or exercise	14 (36.8%)
Take a drive	14 (36.8%)
Meditate or take deep breaths	12 (31.6%)
Spend time with a pet	11 (28.9%)
Read	10 (26.3%)
Spend time with a loved one	10 (26.3%)
Eat a snack	9 (23.7%)
Go to the mall	4 (10.5%)
Look at pictures of my loved ones	4 (10.5%)
Play a video game	3 (7.9%)
Other (respondents wrote in research, spend time with my kids)	2 (7.9%)

^1^ A total of 38 respondents selected at least one pleasurable activity. The percentages exceeded 100% because the respondents could select multiple pleasurable activities.

## Data Availability

The data presented in this study are available from the corresponding author upon request due to privacy reasons.
